# Exposure to Environmentally Relevant Concentrations of Polystyrene Microplastics Increases Hexavalent Chromium Toxicity in Aquatic Animals

**DOI:** 10.3390/toxics10100563

**Published:** 2022-09-26

**Authors:** Jaehee Kim, Md. Niamul Haque, Somyeong Lee, Do-Hee Lee, Jae-Sung Rhee

**Affiliations:** 1 Department of Marine Science, College of Natural Sciences, Incheon National University, Incheon 22012, Korea; 2 Research Institute of Basic Sciences, Incheon National University, Incheon 22012, Korea; 3 Yellow Sea Research Institute, Incheon 22012, Korea

**Keywords:** chromium toxicity, polystyrene microplastics, aquatic models, life endpoint

## Abstract

The prevalence of hexavalent chromium [Cr(VI)] and microplastics (MPs) is ubiquitous and is considered a threat to aquatic biota. MPs can act as a vector for waterborne metals; however, the combined effects of Cr(VI) and MPs on aquatic organisms are largely unknown. In this study, aquatic model animals, such as rotifers (*Brachionus calyciflorus* and *B. plicatilis*), water fleas (*Daphnia magna*), amphipods (*Hyalella azteca*), polychaetes (*Perinereis aibuhitensis*), and zebrafish (*Danio rerio*) were exposed to environmental concentrations (1, 10, and 100 particles L^−1^) of 1 μm polystyrene MPs alone, Cr(VI) alone, or Cr(VI) combined with MPs. Following exposure, the potential effects were measured by analyzing basic life endpoints (e.g., survival rate and growth). A significant response to MPs alone was not observed in all animals. However, MPs combined with Cr(VI) concentration-dependently increased Cr(VI) toxicity in two rotifer species. The survival rate of water fleas was significantly reduced upon exposure to Cr(VI) + MPs (100 particles L^−1^) compared with exposure to Cr(VI) alone, and significantly decreased the number of offspring. Although there was no significant effect on the body length of the amphipod, concentration-dependent decreases in their survival rates were observed. In contrast, no significant change was found in the survival rate of polychaetes; however, their burrowing ability was inhibited by Cr(VI) + MPs (100 particles L^−1^). Further, larval mortality was increased in response to Cr(VI) + MPs (100 particles L^−1^) in zebrafish. Taken together, the findings suggest that MPs can exacerbate Cr(VI) toxicity, even at environmental levels.

## 1. Introduction

In aquatic and terrestrial environments, chromium (Cr) mainly exists in two oxidation states, trivalent chromium [Cr(III)] and hexavalent chromium [Cr(VI)], which are released from diverse natural and anthropogenic sources, including rocks, welding, leather tanning, chrome plating, chrome pigmenting, manufacturing of various alloys, and automobile emissions [1,2]. Although the environmental concentration of Cr is relatively low (<1 μg L^−1^) in groundwater, its level is markedly higher in industrial areas. In fact, up to 84 μg L^−1^ Cr has been measured in the surface waters of the USA, while 0.2 to 44 μg L^−1^ has been recorded in those of central Canada [3,4]. Cr(VI) exerts greater toxicity than Cr(III), as water-soluble Cr(VI) readily penetrates the cell membrane via non-specific anion carriers when absorbed in the tissue of organisms [2,5]. Cr(VI) has been found to be highly genotoxic and carcinogenic in vitro and in vivo, including in mammalian systems [6,7]. As one of the major mechanisms for its toxicity, Cr(VI) triggers the production of reactive nitrogen species (RNSs) and reactive oxygen species (ROSs), subsequently causing the initiation of genomic instability [8]. In fish, Cr(VI) induces developmental malformation, reproduction failure, and immunotoxicity [1,9,10].

The global production of plastics has increased per annum and has received international attention for its potential impacts on ecosystems [11]. Nano- and micro-sized plastic debris, referred to as nanoplastics (NPs) and microplastics (MPs), can be transported to the aquatic environment through mechanical and/or photochemical environmental factors (e.g., waves and weathering) [12,13]. Primary plastics are pieces manufactured for diverse commercial purposes, such as cosmetics and personal care products, synthetic textiles, industrial manufacturing, and fishing activity [14,15]. Plastics can be degraded into smaller pieces as secondary plastics by numerous anthropogenic and/or environmental factors in aquatic environments [13,15]. Both primary and secondary plastics contribute to global NP/MP pollution in aquatic environments due to their persistency and buoyancy. MPs have been detected worldwide in oceanic compartments, including surface water [16,17], deep sea, seabed [18,19], and shorelines [20], as well as polar regions and remote islands [21,22]. In marine ecosystems, concentrations of MPs have been reported to be up to 100,000 particles m^−3^ [23]. Ingestion of MPs and their temporal bioconcentration and trophic transfer from primary producers to consumers has been extensively observed in numerous aquatic organisms [24,25,26,27]. The recognized harmful effect of MPs on aquatic organisms includes reduced feeding, gut blockage, suffocation, mortality, growth retardation, reproduction failure, and delayed ovulation, as well as biochemical effects such as endocrine disruption, immune modulation, and oxidative stress [24,25,26,27].

Due to their high fugacity capacity and large surface area, MPs possess a high affinity toward metals. Considering the broad presence of hexavalent chromium and MPs in the environment, these two contaminants can interact with each other. In addition to the direct threats of these chemicals, adsorption of Cr(VI) by MPs may alter the toxic potential and bioavailability of these contaminants [22], called the “Trojan horse” effect. Adsorption of Cr(VI) was observed on the surface of MPs, where the oxyanions (e.g., HCrO_4_^−^ and CrO_4_^2−^) of Cr(VI) and positive regions of plastic interacted in aquatic environments [28]. The toxic potency of Cr(VI) and MPs to organisms can be synergistic or antagonistic under different abiotic factors, such as concentration, exposure period, pH, salinity, alkalinity, hardness, and temperature; and biotic factors, including developmental stage, organs, and species [10,29]. In zebrafish, both synergistic [30] and antagonistic effects [31] have been suggested to result from the combined exposure to MPs and copper. In zebrafish embryos, combined exposure to MPs and cadmium caused synergistic detrimental effects by reducing heart rate and inducing growth retardation. However, exposure was also found to decrease mortality as an antagonistic effect owing to the strong absorption of Cd on MPs compared to single exposure [32]. The exposure of MPs to five metals (As, Cd, Cu, Pb, and Zn) increased metal bioconcentration, resulting in synergistic detrimental effects on the antioxidant defense system and cholinergic system in a marine mysid [33]. The toxicity of MPs or Cr(VI) alone is well established; however, information on their combined toxicity is scarce. Thus, their combined effect on different trophic levels is important, as the ecosystems contain numerous animal taxa and exert different sensitivities [26].

In this study, the combined effects of Cr(VI) and MPs were assessed in representative aquatic models, including two rotifers (*Brachionus calyciflorus* and *Brachionus plicatilis*), the water flea *Daphnia magna*, the amphipod *Hyalella azteca*, the polychaete *Perinereis aibuhitensis*, and zebrafish (*Danio rerio*), which have been extensively used to understand the potential effect of environmental pollutants. In general, these animals are suitable model organisms in ecotoxicological study owing to their high sensitivity, ease of cultivation, rapid population growth, and ubiquitous nature in marine and freshwater ecosystems [5]. It is of note that the animals selected in this study can represent partial trophic levels from prey (e.g., zooplankton) to scavengers (e.g., polychaetes) and consumers (e.g., fish). Numerous MP types and their sizes have been confirmed to be ingested by these animals, such as rotifers [34,35], water fleas [36,37,38], amphipods [39,40,41], polychaetes [23,42,43,44,45], and zebrafish [46,47].

As environmental concentrations of MPs in marine ecosystems range from 0.04 particles m^−3^ (Portuguese coast) [17] to markedly higher concentrations, such as in Hong Kong (51–27,909 particles m^−3^) [48], the North Yellow Sea of China (545 ± 282 particles m^−3^) [49], the Yangtze estuary (4137.3 ± 2461.5 particles m^−3^) [50], the southeastern coastline of South Africa (257.9 ± 53.36–3308 ± 1449 particles m^−3^) [51], and Cape Cod to the Caribbean in the Atlantic (6.06 × 10^3^–8.32 × 10^5^ particles m^−3^) [52], the concentration range employed in this study was set to cover environmental and relatively higher concentrations. Thus, the potential effects of the combination of Cr(VI) and MPs were estimated in response to environmental concentrations (1, 10, and 100 particles L^−1^) of MPs alone, Cr(VI) alone, and Cr(VI) combined with different concentrations of MPs by measuring endpoints established for each animal, such as survival, growth, offspring, and other physiological responses.

## 2. Materials and Methods

### 2.1. Chemicals and Microplastics

Analytical-grade sodium dichromate dihydrate [Na_2_Cr_2_O_7_·2H_2_O; Cr(VI)] was purchased from Sigma-Aldrich, Inc. (St. Louis, MO, USA). Aqueous solutions were prepared using deionized water (R ≥ 18.0 MΩ·cm) from a Barnstead water purification system (Barnstead, Newton, MA, USA).

For the MPs experiment, 1 μm of non–functionalized polystyrene microbeads were purchased as aqueous suspensions in distilled water (2.5 mg mL^−1^) from Sigma-Aldrich, Inc. According to the manufacturer, the mean diameter of the particle was 1 μm, and the calibrated particle diameter was 1.00–1.20 μm. The suspension was gently mixed using a vortex before dilution and use. No surfactant was used during the dilution procedure. Working solutions at designated nominal concentrations were prepared by diluting a stock solution in freshwater or filtered artificial seawater (ASW; 30 practical salinity unit; TetraMarine Salt Pro, Tetra, Cincinnati, OH, USA). The potential effects of environmental concentrations (1, 10, and 100 particles L^−1^) of MPs, Cr(VI), and the combined effects of Cr(VI) and different concentrations of 1 μm MPs on each animal were monitored.

### 2.2. Rotifer Toxicity Test

Two rotifer species, *Brachionus calyciflorus* and *Brachionus plicatilis*, were employed as representative models for freshwater and brackish/marine environments, respectively. Stock cultures for the two rotifers were maintained under static–renewed conditions with a light:dark (L:D) photoperiod of 14:10 h. The freshwater rotifer *B. calyciflorus* was cultured in artificial Environmental Protection Agency (EPA) freshwater medium (60 mg CaSO_4_·H_2_O L^−1^, 4 mg KCl L^−1^, 123 mg of MgSO_4_ L^−1^, 96 mg NaHCO_3_ L^−1^) [53] dissolved in deionized water (pH 7.5, 25 °C; Millipore, Bedford, MA, USA). The brackish/marine *B. plicatilis* was reared at 30 psu in filtered ASW through 0.45 μm pore size cellulose nitrate membrane filter. Both species were fed a suspension of the green alga *Chlorella vulgaris* (3 × 10^6^ cells mL^−1^) every three days.

The acute toxicity test for measuring the median lethal concentration (LC50) value was conducted based on the standard protocol established in *B. calyciflorus* and *B. plicatilis* [54,55]. Briefly, 10 neonates (<3 h old) were placed in 1 mL in each of the 4 replicates (a total of 40 neonates per concentration) as part of the control or treated group in 24-well plates (SPL Life Science, South Korea). Thereafter, the 24 h LC50 values for Cr(VI) were measured in each species, with concentrations ranging from 0–100 mg L^−1^ for Cr(VI). Subsequently, additional mortality values were calculated under the same experimental conditions using Cr(VI) with additions of different concentrations of 1 μm MPs (1, 10, and 100 particles L^−1^). For fecundity measurement, three neonates (age < 1 h) were collected and separated into 12-well polystyrene plates (SPL Life Science). The neonates were then exposed to Cr(VI) with different concentrations of MPs, and their fecundity was measured for seven days under a semi-static exposure condition. During the experiment, one-half of the artificial seawater with equivalents of Cr(VI) and MPs was changed every two days, and algal diet was supplied.

### 2.3. Water Flea Toxicity Test

The cladoceran *Daphnia magna* was cultured in a fully defined medium M7 [56] at a rate of 20 daphnids per 800 mL of culture medium (pH 7.5) in a 1 L glass beaker at 20 °C under an L:D photoperiod of 14:10 h. The medium was prepared with distilled water after filtration (Millipore, Bedford, MA, USA). The water fleas were fed once daily with a mixture of the green alga *Chlorella vulgaris* (2 × 10^5^ cells mL^−1^) and baker’s yeast.

An acute toxicity test was conducted based on the standard protocol for *D. magna* [57]. Briefly, twenty neonates (<24 h old) were divided into four groups (25 mL test solution in a 100 mL glass beaker) and exposed to Cr(VI) (0–200 μg L^−1^ at 10 μg L^−1^ intervals) or a combination of Cr(VI) and 1 μm MPs (1, 10, and 100 particles L^−1^) in a static culture condition for 48 h. For the control group, 30 neonates were divided into six groups. The number of dead daphnids was counted after 24 and 48 h.

Reproductive output was monitored as a chronic response to Cr(VI) and the combination of Cr(VI) and 1 μm MPs based on the standard reproduction test protocol for *D. magna* [56]. Exposure doses for each chemical were set within 48 h. The no observed effect concentration (NOEC) value was 12 μg L^−1^ for Cr(VI). Five neonates (<24 h) were individually exposed to each sublethal concentration of Cr(VI) (0, 1, 5, and 10 μg L^−1^) or the combination of Cr(VI) and 1 μm MPs for 24 days in a semi-static culture condition. A daphnid was housed in a 100 mL glass beaker containing 80 mL of test solution. The culture medium was changed with the addition of the same concentration of Cr(VI) and MPs, and the produced offspring were discarded twice weekly. The daphnids were fed once daily. Test solutions were renewed three times per week, and the number of offspring in each container was counted.

### 2.4. Amphipod Chronic Toxicity Test

The amphipod *Hyalella azteca* was cultured in dechlorinated tap water (pH 8.0, hardness = 1.3 mM, alkalinity = 1.0 mM, conductivity = 298 μS cm^−1^). Culture and toxicity tests were performed in an automated aquaculture system at Incheon National University (Incheon, South Korea) at 20 °C under an L:D photoperiod of 16:8 h. The amphipods were fed the algae *Selenastrum capricornutum*, yeast-Cerophyl-Trout Chow^®^ (YCT; Aquatic BioSystem Inc., Fort Collins, CO, USA), and suspended flake (Tetramin diet) mixture once daily.

Toxicity was determined by measuring 28- and 42-day survival based on the amphipod chromic toxicity testing method [53]. For direct comparison of toxicity, nominal concentrations of Cr(VI) (0, 3.125, 6.25, 12.5, 25, and 50 μg L^−1^) and exposure conditions were employed as described in a previous toxicity study conducted on *H. azteca* [58]. In addition to the experimental conditions, survival and body growth were measured for amphipods exposed to Cr(VI) or the combination of Cr(VI) and 1 μm MPs. Seven-day-old amphipods (1.46 ± 0.08 mm) were collected, and individuals were reared in a 300 mL glass beaker containing 200 mL of the test solution. For each concentration, 30 amphipods were exposed without pooling, and their survival was determined as a percentage (%) at days 28 and 42, with measurement of the carapace length (mm) as a growth parameter. During the experiment, one-half of the artificial seawater with equivalents of Cr(VI) and MPs was changed every five days, and the amphipods were fed once daily.

### 2.5. Polychaete Acute Toxicity Test

Approximately 1000 individuals (≈1.49 ± 0.22 g; 70 days after fertilization) of the marine polychaete *Perinereis aibuhitensis* were transferred from the polychaete aquaculture company (Yeosu, South Korea) and reared at the automated aquaculture system of Incheon National University using ASW (TetraMarine Salt Pro) under conditions of a 16:8 h L:D photoperiod, 18 ± 0.5°C, 32 psu, 7 mg L^–l^ DO, and pH 8.0, with trickle flow aeration.

To determine the 96 h LC50 value, 50 polychaetes (n = 10 per each concentration) were exposed to different concentrations of Cr(VI) or the combination of Cr(VI) and 1 μm MPs in a 55 × 45 × 36 cm opaque glass fiber tank (n = 10 per tank; 21stcentury HighTech^®^, Pusan, South Korea) without sediment under the same conditions of polychaete culture. Half of the test solution was renewed daily, and an equivalent concentration of the respective Cr(VI) and MPs was replenished. On a daily basis, dead polychaetes were detected and removed from the test chamber. At the end of the treatment, approximately 1–3% dead polychaetes were recorded in the control and citric acid-treated groups at 96 h. No food was supplied during the toxicity experiment.

The live polychaetas were collected after 96 h of exposure to the LC50 of Cr(VI), its 1/10 value, and the LC50 of Cr(VI) + MPs to determine their burrowing ability based on a previous burrowing assay with *P. aibuhitensis* [59]. Twenty live polychaetes from each treatment were moved to clean ASW for the burrowing assay; a 200 mL glass fiber container filled with ASW and sieved sediment (depth of 5 cm), which is the same as that used for acclimation, was used. Full burrowing was counted every two minutes for 30 min.

### 2.6. Zebrafish Early Life Stage Test

Zebrafish were cultured in an automated aquaculture system with mechanical and biological filters at Incheon National University (Incheon, South Korea). Fish were maintained in the controlled system at 26 ± 0.5 °C with an L:D photoperiod of 14:10 h with continuous aeration. Fish were fed live brine shrimp nauplii bred from dry embryos (<24 h old; SERA Artemia, Salt Lake, UT, USA) twice per day at a rate of 5% of body weight. Water chemical parameters were analyzed once per week using Sera ammonium, nitrite, and nitrate kits (Sera, Heinsberg, Germany).

To directly compare the potential effects of Cr(VI) on the early life stages of zebrafish, we performed an assay conducted in a previous study [9]; this assay originally followed the OECD draft guidelines on fish embryo toxicity (FET) testing [60]. Although the EU Directive 2010/63/EU on the protection of animals used for scientific purposes suggested that the earliest life stages of zebrafish are not defined as protected until the stage of being capable of independent feeding (120 h) [61], our experiment was conducted for 144 h, as we wanted to directly compare our results with that of the previous study performed for 144 h [9]. The concentrations of Cr(VI) were 0, 21, 49, 75, 93, 116, and 120 mg L^−1^ [9]. Subsequently, additional mortalities were calculated under the same experimental conditions using the combination of Cr(VI) and 1 μm MPs.

Zebrafish eggs were sampled within 30 min after mating. After brief washing with water, fertilized eggs were confirmed under a Nikon SMZ25 stereomicroscope (Nikon, Japan). Unfertilized eggs were discarded from the experiment. Seventy-two eggs per each concentration (12 replicates) were collected and distributed individually in 24-well plates (SPL Life Science). Exposure was performed under a semi-static culture condition for 6 days. Almost all embryos hatched in the well plates at approximately 72 h. Embryos, hatched larvae, and mortality were counted daily under a Nikon SMZ25 stereomicroscope (Nikon).

### 2.7. Statistical Analysis

The acute toxicity data (e.g., NOEC and LC50) and corresponding 95% CIs were determined according to probit analysis using ToxRat^®®^ Professional 2.10.3.1 (ToxRat Solutions GmbH, Alsdorf, Germany). All data are presented as mean ± standard deviation (S.D.). Statistical significance was determined using the statistics software package, SPSS (ver. 17.0, SPSS Inc., Chicago, IL, USA). Significant differences in the variables measured among treatments were derived using one-way analysis of variance (ANOVA). A post hoc Tukey HSD test was performed to determine pairwise differences with time and concentration. A type I error probability of *p* < 0.05 was considered statistically significant.

## 3. Results

### 3.1. Effect of Cr(VI) and MP on Rotifer Survival

The 24 h LC50 values for Cr(VI) were 0.3 and 2 mg L^−1^ for *B. calyciflorus* and *B. plicatilis*, respectively. The 7-day population growth of rotifers was not significantly affected by exposure to MPs (1, 10, and 100 particles L^−1^) alone (Figure S1).

**Figure 1:**
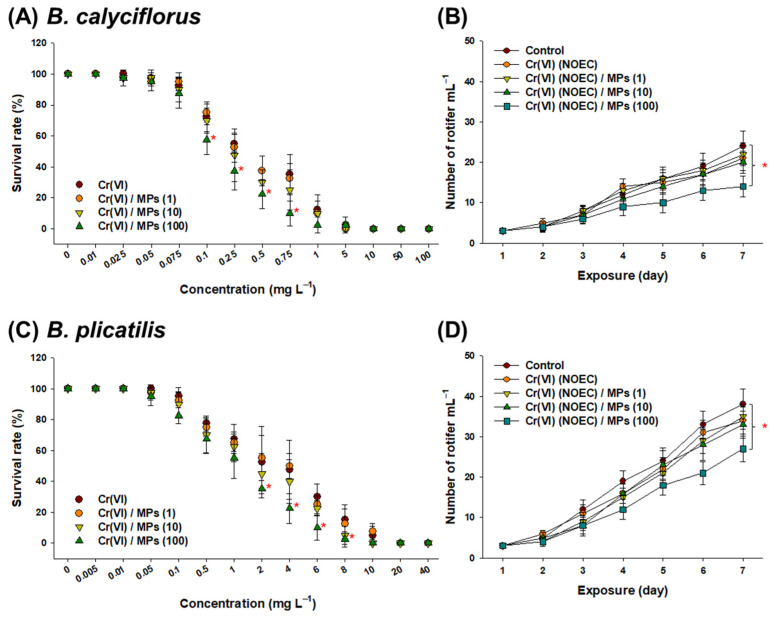
Results of acute toxicity in rotifers. Measurement of the 24 h survival rate of (**A**) *B. calyciflorus* and (**C**) *B. plicatilis* in response to different concentrations of Cr(VI) (0–100 mg L^−1^ for *B. calyciflorus* and 0–40 mg L^−1^ for *B. plicatilis*) and the combination of Cr(VI) and 1 μm MPs (1, 10, and 100 particles L^−1^). The number of (**B**) *B. calyciflorus* and (**D**) *B. plicatilis* during the 7 days of exposure to the NOEC value of Cr(VI) and the combination of Cr(VI) and 1 μm MPs (1, 10, and 100 particles L^−1^). Data are presented as mean ± standard deviation (S.D.) of four replicates (n = 10 per replicate). An asterisk (*) on each concentration or day indicates significant difference between the control and exposure groups (*p* < 0.05).

The survival rate of *B. calyciflorus* was significantly reduced by 0.10–0.75 mg L^−1^ Cr(VI) + MPs (100 particles L^−1^) for 24 h compared to Cr(VI) alone (Figure 1A). The number of rotifers significantly decreased following exposure to the NOEC value of Cr(VI) with MP (100 particles L^−1^) at day 7 compared to the control (*p* < 0.05) (Figure 1B).

The survival rate of *B. plicatilis* significantly decreased when exposed to 2–8 mg L^−1^ Cr(VI) + MPs (100 particles L^−1^) compared to Cr(VI) alone (Figure 1C). Similar to *B. calyciflorus*, the population number of *B. plicatilis* was significantly reduced in response to the NOEC value of Cr(VI) with MPs (100 particles L^−1^) at day 7 (*p* < 0.05) (Figure 1D). The survival rate did not significantly differ following treatment with Cr(VI) + MPs (1 particle L^−1^) and Cr(VI) + MPs (10 particles L^−1^) compared to Cr(VI) alone (*p* > 0.05).

### 3.2. Effect of Cr(VI) and MPs on the Survival and Offspring Production of Water Fleas

**Figure 2:**
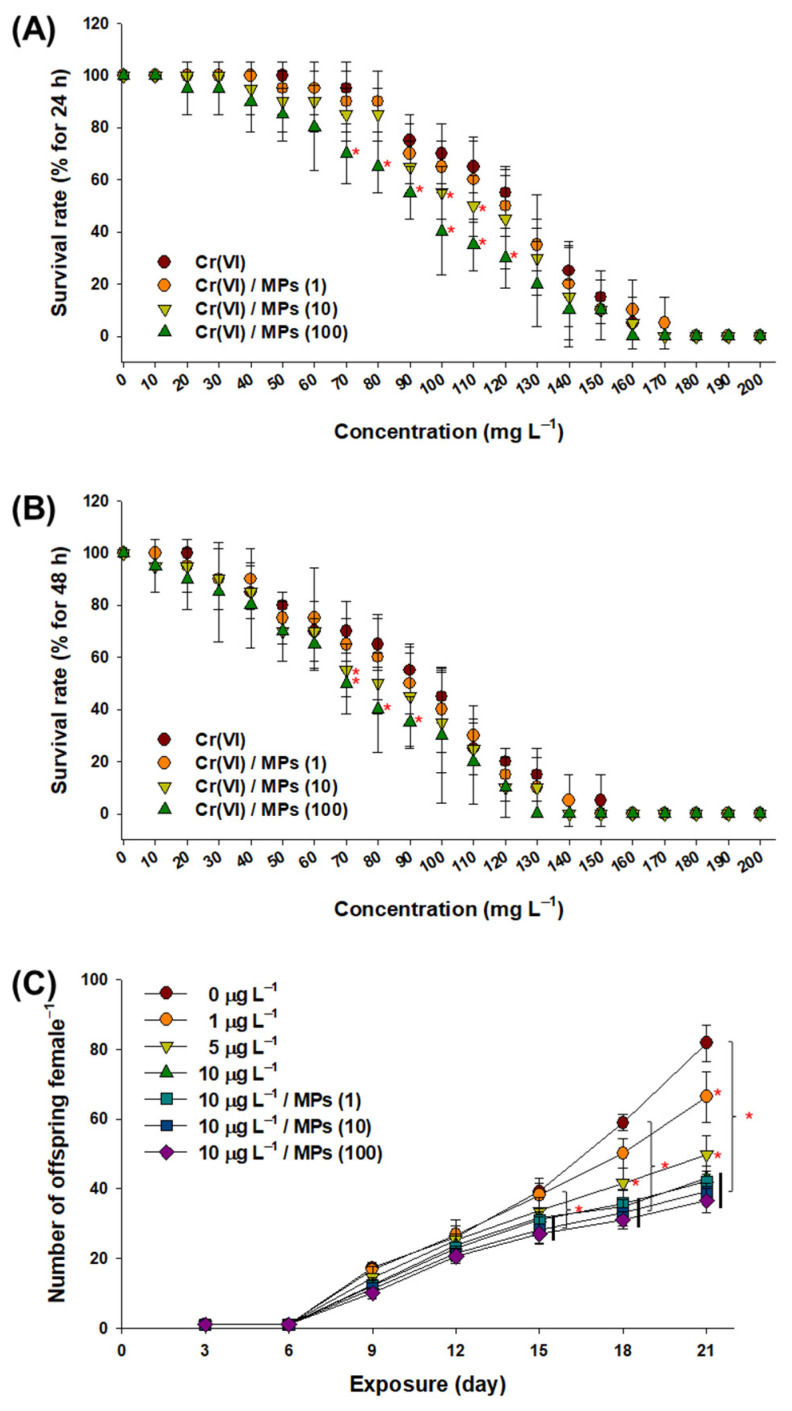
Results of acute toxicity and the chronic reproductive impact of Cr(VI) + MPs on water fleas. Measurement of the survival rate of daphnids in response to Cr(VI) (0–200 μg L^−1^ at 10 μg L^−1^ intervals) and the combination of Cr(VI) and 1 μm MPs (1, 10, and 100 particles L^−1^) at (**A**) 24 h and (**B**) 48 h. (**C**) Number of live offspring per female following 21 days of exposure to sublethal concentrations of Cr(VI) (0–10 μg L^−1^) and the combination of Cr(VI) and 1 μm MPs (1, 10, and 100 particles L^−1^). Data are presented as mean ± standard deviation (S.D.) of three groups. An asterisk (*) indicates significant difference between the exposed groups for each concertation or day (*p* < 0.05).

The LC50 value of Cr(VI) was 115 μg L^−1^ at 24 h. The 21-day population growth of water fleas was not significantly affected by exposure to MPs (1, 10, and 100 particles L^−1^) alone (Figure S2). However, the 21-day population growth of water fleas exposed to 100 and 110 mg L^−1^ Cr(VI) + MPs (10 particles L^−1^) and 70–120 mg L^−1^ Cr(VI) and Cr(VI) + MPs (100 particles L^−1^) significantly decreased compared to those exposed to Cr(VI) alone (*p* < 0.05) (Figure 2A).

At 48 h, the LC50 value of Cr(VI) was 81 μg L^−1^. After 48 h of exposure, a significant decrease in survival rate was found in the 70 mg L^−1^ Cr(VI) + MPs (10 particles L^−1^) group and 70–90 mg L^−1^ Cr(VI) + MPs (100 particles L^−1^) group compared to the Cr(VI) alone group (*p* < 0.05) (Figure 2B).

The offspring numbers of female water fleas exposed to 1 and 5 µg L^−1^ of Cr(VI) were significantly decreased at day 21 compared to those of the control (*p* < 0.05) (Figure 2C). Significant decreases in offspring numbers were also observed in response to 10 μg L^−1^ Cr(VI) alone and the groups administered both Cr(VI) and MPs (1, 10, and 100 particles L^−1^) at days 15, 18, and 21 (*p* < 0.05). There was no significant difference between the 10 μg L^−1^ Cr(VI) alone group and the groups administered both Cr(VI) and MPs (*p* > 0.05).

### 3.3. Effect of Cr(VI) and MPs on the Survival Rate and Growth of Amphipods

**Figure 3:**
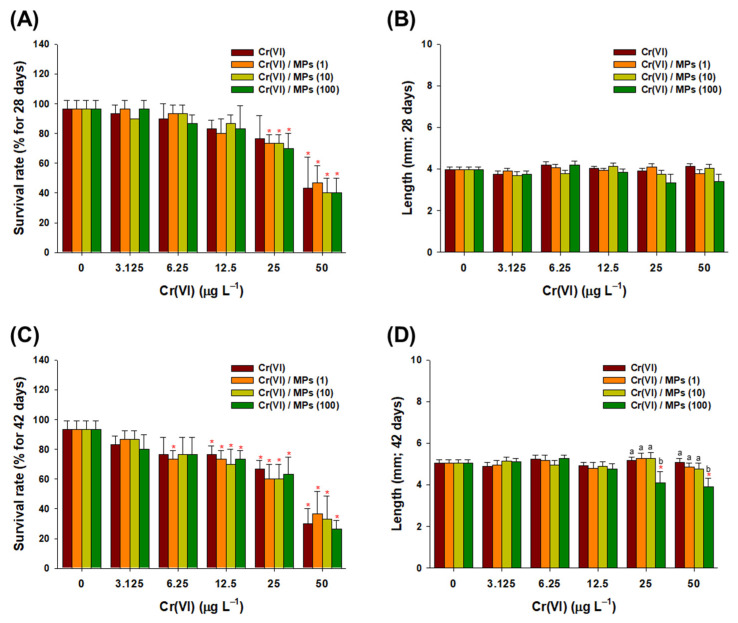
Toxicity test results for the amphipod *Hyalella azteca*. Measurement of the (**A**) survival rate and (**B**) growth (mm) of *H. azteca* in response to Cr(VI) (0–50 μg L^−1^) and the combination of Cr(VI) and 1 μm MPs (1, 10, and 100 particles L^−1^) at day 28. Measurement of the (**C**) survival rate and (**D**) growth (mm) of *H. azteca* in response to Cr(VI) (0–50 μg L^−1^) and the combination of Cr(VI) and 1 μm MPs (1, 10, and 100 particles L^−1^) at day 42. Data are presented as mean ± standard deviation (S.D.). Letters indicate significant difference between the exposed groups on each concentration of Cr(VI) (*p* < 0.05). An asterisk (*) indicates significant difference between the control and exposure groups (*p* < 0.05).

Overall, dose-dependent decreases in survival rate were observed in response to Cr(VI) alone and the combination of Cr(VI) and MPs for 28 and 42 days. However, MPs alone did not have a significant effect on the survival and growth of amphipods at days 28 and 42 (data not shown). MPs were found to have significant additional effects on the survival rate of amphipods following 28-day exposure to 25 and 50 µg L^−1^ Cr(VI) (*p* < 0.05) (Figure 3A). Similarly, at day 42, significantly lower survival rates were observed following exposure to 6.25 µg L^−1^ Cr(VI) and MPs (1 particle L^−1^) and 12.5, 25, and 50 µg L^−1^ Cr(VI) compared to the control (*p*  <  0.05) (Figure 3C). There was no significant change in the growth parameter after 28 days of exposure (*p*  >  0.05) (Figure 3B); however, significant growth inhibition was detected in response after 48 days of exposure (*p*  <  0.05) (Figure 3D).

### 3.4. Effect of Cr(VI) and MPs on the Survival and Burrowing Capacity of Polychaetes

**Figure 4:**
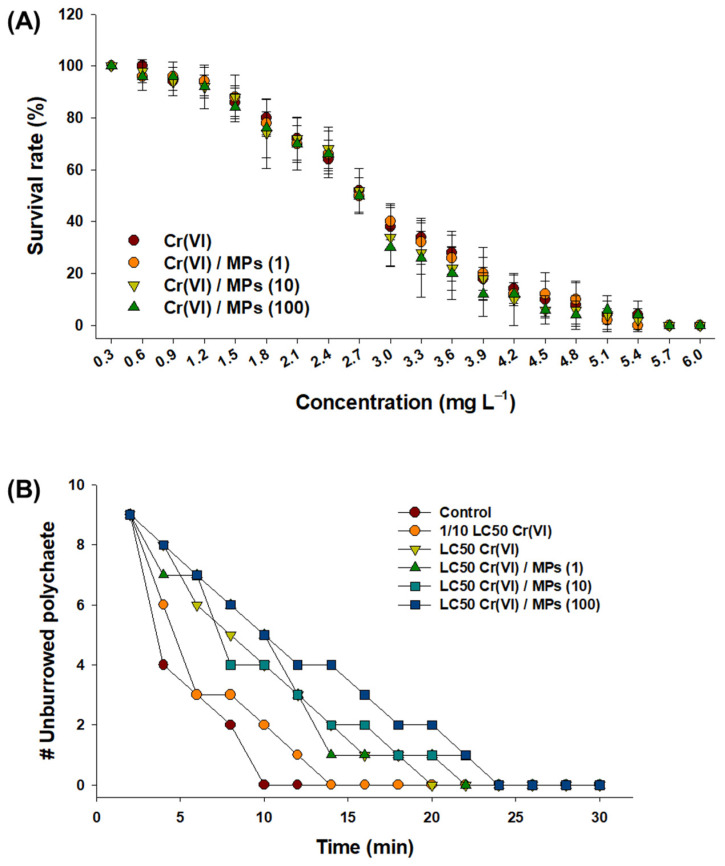
Results of acute toxicity and the burrowing behavior of the polychaete *P. aibuhitensis.* (**A**) Measurement of the 96-h survival rate of *P. aibuhitensis* in response to different concentrations of Cr(VI) (0.3–6.0 mg L^−1^) and the combination of Cr(VI) and 1 μm MPs (1, 10, and 100 particles L^−1^). Data are presented as mean ± standard deviation (S.D.) of five replicates (n = 10 per replicate). (**B**) Effects of the LC50 concentration of Cr(VI), its 1/10 LC50 value, and the combination of Cr(VI) and 1 μm MPs (1, 10, and 100 particles L^−1^) on the burrowing behavior of *P. aibuhitensis*. Data are presented as mean ± standard deviation (S.D.) of 20 individuals.

There were no significant differences in the response of *P. aibuhitensis* to Cr(VI) alone and Cr(VI) + MPs (*p* > 0.05) (Figure 4A). The 100% mortality of polychaete was measured at 5.7 mg L^−1^ for the different treatment groups. The burrowing ability of polychaete was delayed for those exposed to the LC50 of Cr(VI) alone and Cr(VI) + MPs (Figure 4B). No significant difference in burrowing ability was observed after exposure to MPs alone (1, 10, and 100 particles L^−1^) (Figure S3). All polychaetes in the control and 1/10 LC50 Cr(VI)-exposed groups burrowed before 15 min, whereas those in the group exposed to the LC50 of Cr(VI) alone and Cr(VI) + MPs burrowed from 20 min.

### 3.5. Effect of Cr(VI) and MPs on the Survival of Zebrafish Embryos and Larvae

**Figure 5:**
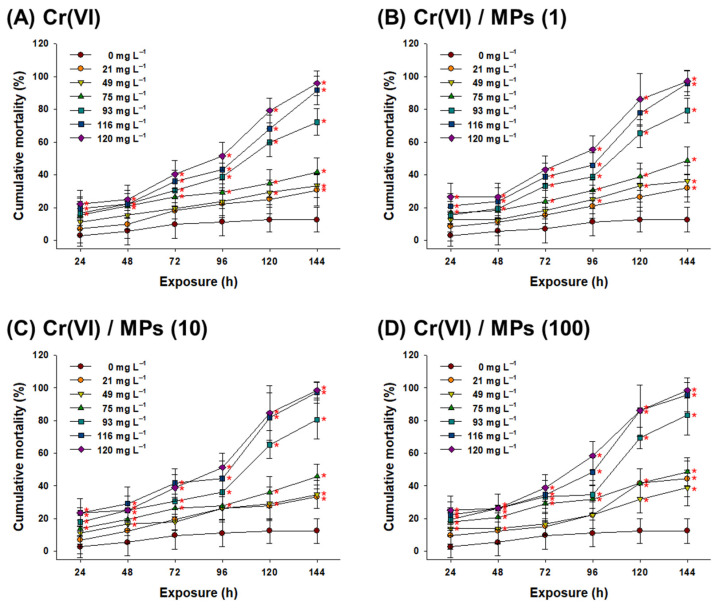
Results of acute toxicity in zebrafish. Cumulative mortality of zebrafish embryos and larvae in response to different concentrations of (**A**) Cr(VI), (**B**) Cr(VI) + 1 μm MPs (1 particle L^−1^), (**C**) Cr(VI) + 1 μm MPs (10 particles mL^−1^), and (**D**) Cr(VI) + 1 μm MPs (100 particles L^−1^) for 144 h. Data are presented as mean ± standard deviation (S.D.) of twelve replicates (n = 6 per replicate). An asterisk (*) on each concertation or day indicates a significant difference between the control and exposure groups (*p* < 0.05).

A significant concentration-dependent increase in mortality was found for zebrafish after Cr(VI) treatment for 144 h (*p* < 0.05) (Figure 5A). MPs alone (1, 10, and 100 particles L^−1^) had no significant effect on the survival of zebrafish at 144 h (Figure S4). At the end of the 144 h treatment period, all groups showed statistical significance compared to the control. Furthermore, a similar mortality rate was observed at the embryonic stage (before 72 h) in the Cr(VI) alone and Cr(VI) + MP groups, while a slight increase in mortality was observed after the transition point (72–96 h) from embryo to larvae (Figure 5A–D). At the end of the 144 h treatment period, higher mortality was measured in response to 93 (83%), 116 (96%), and 120 mg L^−1^ (99%) Cr(VI) + MPs (100 particles L^−1^) compared to 93 (72%), 116 (92%), and 120 mg L^−1^ (96%) Cr(VI) alone (Table 1, Figure 5D).

## 4. Discussion

The species-specific response of aquatic organisms to Cr(VI) + MPs was examined in this study. When the highest concentration of MPs was employed, a significant increase in the mortality of rotifers, water fleas, and amphipods was found, suggesting a synergistic detrimental effect of MPs on Cr(VI) toxicity. Although many studies revealed that MPs themselves can induce mortality through severe digestive system dysfunction, gut blockage, and feeding inhibition at extremely high concentrations, such direct damage was negligible in this study; this is because each aquatic animal was exposed to environmentally relevant concentrations of MPs. Synergistic toxicological effects can be manifested by higher attachment of Cr(VI) on the hydrophobic surface of MPs via sorption and a subsequent long preservation in internal organs [62,63,64,65]. The mechanism of Cr(VI) toxicity involves its direct entrance into the cell through anionic channels (e.g., HPO_4_^2−^ and SO_4_^2−^) and/or phagocytosis and production of strongly oxidized pentavalent and tetravalent chromium, resulting in the generation of oxygen-free radicals that are responsible for oxidative stress and damage to DNA and proteins [66]. The attached Cr(VI) can be released from MPs after the adsorption bond in the digestive tract is weakened, and the released Cr(VI) would induce decreased food consumption and subsequent energy loss, ultimately leading to death [14]. Thus, the cumulative adverse impact of Cr(VI) and MPs at the metabolic process could be responsible for the increased mortality of the animals tested. The number of rotifer and water flea offspring was significantly lowered by the presence of MPs, suggesting their synergistic maternal effect on the second generation. Determining the sole effect of MPs or their combined toxicity with Cr(VI) on embryo development and endocrine systems (e.g., ecdysone metabolism) would be an interesting future study.

The exposure duration-dependent growth retardation in amphipods clearly suggested the synergistic detrimental effects of Cr(VI) + MPs, as significant inhibition was only observed at the highest MP concentration. Such findings imply that prolonged exposure to Cr(VI) + MPs caused critical inhibition of amphipod growth. The aging process changes the surface characteristics of MPs (i.e., reduction of their hydrophobic nature with the increase in carboxyl and ketone groups) as well as metal sorption rate with biofilm development [67]. Based on the results obtained after 42 days of exposure, MPs had a chronic effect on *H. azteca* growth, as no significant changes were observed at day 28. MPs may become attractive food items to organisms through the aging process. The formation of biofilms increases the metal concentration on the surface of the MPs, which can influence metal bioavailability and induce toxicity. A study using the amphipod *H. azteca* reported the elevation of polyethylene MP ingestion owing to an increase in the exposure period [39]. Thus, amphipods can consume a higher quantity of MPs and the combined effects of Cr(VI) and MPs during chronic exposure may induce growth inhibition by modulating the molting processes and/or exoskeleton development [33,39,68,69,70].

Acute toxicity testing and measurement of burrowing capacity have been used extensively as biomarkers for estimating health status in polychaetes [59,71]. The ingestion of MPs by species-specific feeding strategies was reported in polychaetes [23,42,43,45]. Based on the survival rate results, MPs had no substantial effect on Cr(VI) toxicity for 96 h in *P. aibuhitensis*. In general, polychaetes are deposit feeders. Thus, an excellent excretion system has been developed to remove ingested sandy or muddy particles in polychaetes, regardless of their selective or non-selective feeding patterns. Thus, a short exposure period (96 h) could be the reason for the lack of a substantial toxic impact. In the lugworm, *Arenicola marina*, 4-week exposure to polyvinylchloride MPs (130 µm) induced feeding activity inhibition, energy reservation reduction, a long gut residence period, and inflammation, and finally affected survival rate [23]. In the present study, one of the acute effects was observed, as the burrowing ability was reduced in response to Cr(VI) + MPs. A delay in burrowing capacity upon exposure to exogenous chemicals indicates the neurotoxic potential in polychaetes [59]. Ingested MPs may release more Cr(VI) during their movement in the digestive system, as the LC50 value of Cr(VI) itself retarded the burrowing activity in the absence of MPs. Strong sorption of xenobiotics on the surface of MPs had negative effects on the lugworm, *A. marina* (e.g., reduced ability of coelomocytes to remove pathogenic bacteria and the ability of worms to engineer sediments, induction of mortality and susceptibility to oxidative stress) [42]. Our results also revealed that the burrowing kinetics were markedly affected by the concentration of MPs (100 particles L^−1^). The concentration of MPs can be an important modulator of burrowing capacity. Increasing the concentration of polystyrene NPs was found to significantly modulate the behavior and regeneration capacity of *P. aibuhitensis* [44].

Zebrafish have been widely used in ecotoxicological research to assess the potential threats posed by exogenous compounds in aquatic environments [9,29]. In this study, a Cr(VI) concentration-dependent increase in mortality was observed in zebrafish embryos and larvae, suggesting its uptake and accumulation at relatively higher concentrations. Similar concentration-dependent increases in mortality were observed for six days in the early life stages and in adult zebrafish exposed to waterborne Cr(VI) [9]. The chorion is an acellular envelope in embryos that acts as a barrier, blocking the entrance of the chemical and protecting the embryos. Cr(VI) induced toxicity during the embryonic stage (before 72 h), suggesting direct penetration of Cr(VI) through the embryo chorion. However, MPs might be blocked by the chorion, as no significant difference was found between the Cr(VI) alone and Cr(VI) + MPs groups. Dechorionation may contribute to the intensification of Cr(VI)-triggered mortality due to direct ingestion and exposure to the large surface area on the larval body [29,72,73]. Mortality would be caused by the induction of severe necrosis in gill tissue (affecting O_2_ uptake and respiration), alterations in locomotor behavior, and hypersecretion of mucus from the whole body, which were reported previously in mosquito fish exposed to Cr(VI) [74]. In zebrafish larvae, 100 particles L^−1^ MPs enhanced Cr(VI) toxicity. MPs can increase the sorption of Cr(VI) on their surface, consequently reducing metal biotransformation and elevating the bioconcentration of Cr(VI) [75]. MPs can also intensify the accumulation and toxicity of cadmium in zebrafish [46]. Previously, a significant increase in mortality was observed in zebrafish after 14 days of exposure to copper (125 mg L^−1^) + MPs [47].

## 5. Conclusions

In conclusion, combined exposure to Cr(VI) and a relatively high concentration of MPs (100 particles L^−1^) had deleterious impacts on aquatic animals. Increased ingestion, bioconcentration, and bioavailability of Cr(VI) in organisms via MPs might be associated with elevated Cr(VI) toxicity. The toxic effect of Cr(VI) on the growth, offspring, and survival of organisms at different trophic levels indicates its detrimental consequences for the population structure of various organisms and the food web function in the presence of MPs. This study encourages a better understanding of the underlying potential effects of environmentally relevant concentration of MPs on metals. Whether dietary Cr(VI) and MPs potentially act as biological and cellular function disruptors and how Cr(VI) and MPs interfere with the physiological functioning of the exposed animals should be further evaluated.

## Figures and Tables

**Table 1:** Cumulative mortality of zebrafish embryos and larvae measured in response to different concentrations of Cr(VI) with 1 μm MPs (1, 10, and 100 particles L^−1^) for 144 h.

MPs Conc. (Particles L^−1^)	Cr(VI) Conc. (mg L^−1^)	Cumulative Mortality (%)					
		24 h	48 h	72 h	96 h	120 h	144 h
0	0	3	6	10	11	13	13
	21	7	10	18	22	25	31
	49	11	15	19	24	29	33
	75	15	21	26	29	35	42
	93	17	22	31	39	60	72
	116	19	22	36	43	68	92
	120	22	25	40	51	79	96
1	0	3	6	7	11	13	13
	21	8	11	15	21	26	32
	49	13	13	18	25	33	36
	75	17	18	24	31	39	49
	93	15	19	33	39	65	79
	116	21	24	39	46	78	96
	120	26	26	43	56	86	97
10	0	3	6	10	11	13	13
	21	7	13	19	26	28	33
	49	11	17	18	26	29	35
	75	14	19	26	28	36	46
	93	18	25	31	36	65	81
	116	24	29	42	44	82	97
	120	24	25	39	51	85	99
100	0	3	6	10	11	13	13
	21	10	13	15	22	42	44
	49	14	14	17	22	32	39
	75	18	21	29	32	42	49
	93	19	26	33	35	69	83
	116	22	26	35	49	86	96
	120	25	26	39	58	86	99

## Data Availability

Data can be made available upon reasonable request.

## Supplementary Materials

The following supporting information can be downloaded at: https://www.mdpi.com/article/10.3390/toxics10100563/s1, Figure S1: The number of rotifers upon MPs alone; Figure S2: The number of daphnids upon MPs alone; Figure S3: Burrowing activity of polychaete upon MPs alone; Figure S4: Mortality of zebrafish upon MPs alone.

## References

[1] Velma V., Vutukuru S.S., Tchounwou P.B. (2009). Ecotoxicology of hexavalent chromium in freshwater fish: A critical review. Rev. Environ. Health.

[2] DesMarias T.L., Costa M. (2019). Mechanisms of chromium-induced toxicity. Curr. Opin. Toxicol..

[3] EPA (1987). Health Advisory: Chromium.

[4] WHO (2003). Chromium in Drinking Water. Background Document for Development of WHO Guidelines for Drinking Water Quality.

[5] Kim B.-M., Kim B., Nam S.-E., Eom H.-J., Lee S., Kim K., Rhee J.-S. (2022). Reductive transformation of hexavalent chromium in ice decreases chromium toxicity in aquatic animals. Environ. Sci. Technol..

[6] Pourahmad J., Peter J.B., Jokar F., Daraei B. (2003). Carcinogenic metal induced sites of reactive oxygen species formation in hepatocytes. Toxicol. Vitr..

[7] Krumschnabel G., Nawaz M. (2004). Acute toxicity of hexavalent chromium in isolated teleost hepatocytes. Aquat. Toxicol..

[8] Henderson J.D., Filice F.P., Li M.S., Ding Z. (2017). Tracking live-cell response to hexavalent chromium toxicity by using scanning electrochemical microscopy. ChemElectroChem.

[9] Domingues I., Oliveira R., Lourenço J., Grisolia C.K., Mendo S., Soares A.M.V.M. (2010). Biomarkers as a tool to assess effects of chromium (VI): Comparison of responses in zebrafish early life stages and adults. Comp. Biochem. Physiol. C Toxicol. Pharmacol..

[10] Aslam S., Yousafzai A.M. (2017). Chromium toxicity in fish: A review article. J. Entomol. Zool. Stud..

[11] Geyer R., Jambeck J.R., Law K.L. (2017). Production, use, and fate of all plastics ever made. Sci. Adv..

[12] Frias J.P.G.L., Nash R. (2019). Microplastics: Finding a consensus on the definition. Mar. Pollut. Bull..

[13] Koelmans A.A., Mohamed Nor N.H., Hermsen E., Kooi M., Mintenig S.M., De France J. (2019). Microplastics in freshwaters and drinking water: Critical review and assessment of data quality. Water Res..

[14] Jiang X., Li M., He D., Luo Y. (2020). Interaction of microplastics and heavy metals: Toxicity, mechanisms, and environmental implications. The Handbook of Environmental Chemistry.

[15] González-Pleiter M., Tamayo-Belda M., Pulido-Reyes G., Amariei G., Leganés F., Rosal R., Fernández-Piñas F. (2019). Secondary nanoplastics released from a biodegradable microplastic severely impact freshwater environments. Environ. Sci. Nano.

[16] Song Y.K., Hong S.H., Eo S., Jang M., Hang G.M., Isobe A., Shim W.J. (2018). Horizontal and vertical distribution of microplastics in Korean coastal waters. Environ. Sci. Technol..

[17] Kwon O.Y., Kang J.-H., Hong S.H., Shim W.J. (2020). Spatial distribution of microplastic in the surface waters along the coast of Korea. Mar. Pollut. Bull..

[18] Galgani F., Leaute J.P., Moguedet P., Souplet A., Verin Y., Carpentier A., Goraguere H., Latrouitee D., Andralf B., Cadiou Y. (2000). Litter on the sea floor along European coasts. Mar. Pollut. Bull..

[19] Kane I.A., Clare M.A. (2019). Dispersion, accumulation, and the ultimate fate of micro-plastics in deep-marine environments: A review and future directions. Front. Earth Sci..

[20] Barnes D.K., Galgani F., Thompson R.C., Barlaz M. (2009). Accumulation and fragmentation of plastic debris in global environments. Philos. Trans. R. Soc. Lond. B Biol. Sci..

[21] Thompson R.C., Olsen Y., Mitchell R.P., Davis A., Rowland S.J., John A.W.G., McGonigle D., Russell A.E. (2004). Lost at sea: Where is all the plastic?. Science.

[22] Wang F., Wong C.S., Chen D., Lu X., Wang F., Zeng E.Y. (2018). Interaction of toxic chemicals with microplastics: A critical review. Water Res..

[23] Wright S.L., Thompson R.C., Galloway T.S. (2013). The physical impacts of microplastics on marine organisms: A review. Environ. Pollut..

[24] Setälä O., Fleming-Lehtinen V., Lehtiniemi M. (2014). Ingestion and transfer of microplastics in the planktonic food web. Environ. Pollut..

[25] Burns E.E., Boxall A.B. (2018). Microplastics in the aquatic environment: Evidence for or against adverse impacts and major knowledge gaps. Environ. Toxicol. Chem..

[26] Barbosa F., Adeyemi J.A., Bocato M.Z., Comas A., Campiglia A. (2020). A critical viewpoint on current issues, limitations, and future research needs on micro- and nanoplastic studies: From the detection to the toxicological assessment. Environ. Res..

[27] Gouin T. (2020). Toward an improved understanding of the ingestion and trophic transfer of microplastic particles: Critical review and implications for future research. Environ. Toxicol. Chem..

[28] Holmes L.A., Turner A., Thompson R.C. (2014). Interactions between trace metals and plastic production pellets under estuarine conditions. Mar. Chem..

[29] Bhagat J., Zang L., Nishimura N., Shimada Y. (2020). Zebrafish: An emerging model to study microplastic and nanoplastic toxicity. Sci. Total Environ..

[30] Roda J.F.B., Lauer M.M., Risso W.E., Dos Reis Martinez C.B. (2020). Microplastics and copper effects on the neotropical teleost *Prochilodus lineatus*: Is there any interaction?. Comp. Biochem. Physiol. Mol. Integr. Physiol..

[31] Santos D., Felix L., Luzio A., Parra S., Cabecinha E., Bellas J., Monteiro S.M. (2020). Toxicological effects induced on early life stages of zebrafish (*Danio rerio*) after an acute exposure to microplastics alone or co-exposed with copper. Chemosphere.

[32] Zhang R., Wang M., Chen X., Yang C., Wu L. (2020). Combined toxicity of microplastics and cadmium on the zebrafish embryos (*Danio rerio*). Sci. Total Environ..

[33] Eom H.-J., Haque M.N., Lee S., Rhee J.-S. (2021). Exposure to metals premixed with microplastics increases toxicity through bioconcentration and impairs antioxidant defense and cholinergic response in a marine mysid. Comp. Biochem. Physiol. C Toxicol. Pharmacol..

[34] Jeong C.-B., Won E.-J., Kang H.-M., Lee M.-C., Hwang D.-S., Hwang U.K., Zhou B., Souissi S., Lee S.-J., Lee J.-S. (2016). Microplastic size-dependent toxicity, oxidative stress induction, and p-JNK and p-p38 activation in the monogonont rotifer (*Brachionus koreanus*). Environ. Sci. Technol..

[35] Sun Y., Xu W., Gu Q., Chen Y., Zhou Q., Zhang L., Gu L., Huang Y., Lyu K., Yang Z. (2019). Small-sized microplastics negatively affect rotifers: Changes in the key life-history traits and rotifer-phaeocystis population dynamics. Environ. Sci. Technol..

[36] Jemec A., Horvat P., Kunej U., Bele M., Kržan A. (2016). Uptake and effects of microplastic textile fibers on freshwater crustacean *Daphnia magna*. Environ. Pollut..

[37] Ziajahromi S., Kumar A., Neale P.A., Leusch F.D.L. (2017). Impact of microplastic beads and fibers on waterflea (*Ceriodaphnia dubia*) survival, growth, and reproduction: Implications of single and mixture exposures. Environ. Sci. Technol..

[38] Kim J., Rhee J.-S. (2021). Biochemical and physiological responses of the water flea *Moina macrocopa* to microplastics: A multigenerational study. Mol. Cell. Toxicol..

[39] Au S.Y., Bruce T.F., Bridges W.C., Klaine S.J. (2015). Responses of *Hyalellaazteca* to acute and chronic microplastic exposures. Environ. Toxicol. Chem..

[40] Iannilli V., Pasquali V., Setini A., Corami F. (2019). First evidence of microplastics ingestion in benthic amphipods from Svalbard. Environ. Res..

[41] Jamieson A.J., Brooks L.S.R., Reid W.D.K., Piertney S.B., Narayanaswamy B.E., Linley T.D. (2019). Microplastics and synthetic particles ingested by deep-sea amphipods in six of the deepest marine ecosystems on Earth. R. Soc. Open Sci..

[42] Browne M.A., Niven S.J., Galloway T.S., Rowland S.J., Thompson R.C. (2013). Microplastic moves pollutants and additives to worms, reducing functions linked to health and biodiversity. Curr. Biol..

[43] Leung J., Chan K.Y.K. (2018). Microplastics reduced posterior segment regeneration rate of the polychaete *Perinereis aibuhitensis*. Mar. Pollut. Bull..

[44] Silva M.S.S., Oliveira M., Lopéz D., Martins M., Figueira E., Pires A. (2020). Do nanoplastics impact the ability of the polychaeta *Hediste diversicolor* to regenerate?. Ecol. Indic..

[45] Vecchi S., Bianchi J., Scalici M., Fabroni F., Tomassetti P. (2021). Field evidence for microplastic interactions in marine benthic invertebrates. Sci. Rep..

[46] Lu K., Qiao R., An H., Zhang Y. (2018). Influence of microplastics on the accumulation and chronic toxic effects of cadmium in zebrafish (*Danio rerio*). Chemosphere.

[47] Santos D., Félix L., Luzio A., Parra S., Bellas J., Monteiro S.M. (2021). Single and combined acute and subchronic toxic effects of microplastics and copper in zebrafish (*Danio rerio*) early life stages. Chemosphere.

[48] Tsang Y.Y., Mak C.W., Liebich C., Lam S.W., Sze E.T.-P., Chan K.M. (2017). Microplastic pollution in the marine waters and sediments of Hong Kong. Mar. Pollut. Bull..

[49] Zhu L., Bai H., Chen B., Sun X., Qu K., Xia B. (2018). Microplastic pollution in North Yellow Sea, China: Observations on occurrence, distribution and identification. Sci. Total Environ..

[50] Zhao S., Zhu L., Wang T., Li D. (2014). Suspended microplastics in the surface water of the Yangtze estuary system, China: First observations on occurrence, distribution. Mar. Pollut. Bull..

[51] Nel H.A., Froneman P.W. (2015). A quantitative analysis of microplastic pollution along the south-eastern coastline of South Africa. Mar. Pollut. Bull..

[52] Colton J.B., Burns B.R., Knapp F.D. (1974). Plastic particles in surface waters of the Northwestern Atlantic. Science.

[53] EPA (2000). Methods for Measuring the Toxicity and Bioaccumulation of Sediment-Associated Contaminants with Freshwater Invertebrates.

[54] Snell T.W., Persoone G. (1989). Acute toxicity bioassay using rotifers. I. A test for brackish and marine environments with *Brachionus plicatilis*. Aquat. Toxicol..

[55] Snell T.W., Moffat B.D., Janssen C., Persoone G. (1991). Acute toxicity tests using rotifers. IV. Effects of cyst age, temperature, and salinity on the sensitivity of *Brachionus calyciflorus*. Ecotoxicol. Environ. Saf..

[56] OECD (Organisation for Economic Cooperation and Development) (1996). Guidelines for Testing of Chemicals. Proposal for Updated Guideline 211, Daphnia magna Reproduction Test. Revisted Draft Document.

[57] ISO (International Organisation for Standardization) (1989). Water Quality ± Determination of the Mobility of Daphnia magna Straus (Cladocera, Crustacea).

[58] Besser J.M., Brumbaugh W.G., Kemble N.E., May T.W., Ingersoll C.G. (2004). Effects of sediment characteristics on the toxicity of chromium (III) and chromium (VI) to the amphipod, *Hyalella azteca*. Environ. Sci. Technol..

[59] Haque M.N., Nam S.-E., Eom H.-J., Kim S.-K., Rhee J.-S. (2020). Exposure to sublethal concentrations of zinc pyrithione inhibits growth and survival of marine polychaete through induction of oxidative stress and DNA damage. Mar. Pollut. Bull..

[60] OECD (Organisation for Economic Cooperation and Development) (2006). Fish Embryo Toxicity Test. Organization for Economic Co-Operation and Development.

[61] Strähle U., Scholz S., Geisler R., Greiner P., Hollert H., Rastegar S., Schumacher A., Selderslaghs I., Weiss C., Witters H. (2012). Zebrafish embryos as an alternative to animal experiments--a commentary on the definition of the onset of protected life stages in animal welfare regulations. Reprod. Toxicol..

[62] Holmes L.A., Turner A., Thompson R.C. (2012). Adsorption of trace metals to plastic resin pellets in the marine environment. Environ. Pollut..

[63] Luís L.G., Ferreira P., Fonte E., Oliveira M., Guilhermino L. (2015). Does the presence of microplastics influence the acute toxicity of chromium(VI) to early juveniles of the common goby (*Pomatoschistus microps*)? A study with juveniles from two wild estuarine populations. Aquat. Toxicol..

[64] Zhang W., Zhang L., Hua T., Li Y., Zhou X., Wang W., You Z., Wang H., Li M. (2020). The mechanism for adsorption of Cr(VI) ions by PE microplastics in ternary system of natural water environment. Environ. Pollut..

[65] Zhang L., Li Y., Wang W., Zhang W., Zuo Q., Abdelkader A., Xi K., Heynderickx P.M., Kim K.H. (2021). The potential of microplastics as adsorbents of sodium dodecyl benzene sulfonate and chromium in an aqueous environment. Environ. Res..

[66] Stohs S.J., Bagchi D. (1995). Oxidative mechanisms in the toxicity of metal ions. Free Radic. Biol. Med..

[67] Vroom R.J., Koelmans A.A., Besseling E., Halsband C. (2017). Aging of microplastics promotes their ingestion by marine zooplankton. Environ. Pollut..

[68] Murray F., Cowie P.R. (2011). Plastic contamination in the decapod crustacean *Nephrops norvegicus* (Linnaeus, 1758). Mar. Pollut. Bull..

[69] Eom H.-J., Nam S.-E., Rhee J.-S. (2020). Polystyrene microplastics induce mortality through acute cell stress and inhibition of cholinergic activity in a brine shrimp. Mol. Cell. Toxicol..

[70] Lee D.-H., Lee S., Rhee J.-S. (2021). Consistent exposure to microplastics induces age-specific physiological and biochemical changes in a marine mysid. Mar. Pollut. Bull..

[71] Reish D.J., Gerlinger T.V. (1997). A review of the toxicological studies with polychaetous annelids. Bull. Mar. Sci..

[72] Lee W.S., Cho H.-J., Kim E., Huh Y.H., Kim H.-J., Kim B., Kang T., Lee J.-S., Jeong J. (2019). Bioaccumulation of polystyrene nanoplastics and their effect on the toxicity of Au ions in zebrafish embryos. Nanoscale.

[73] Wang W., Ge J., Yu X. (2020). Bioavailability and toxicity of microplastics to fish species: A review. Ecotoxicol. Environ. Saf..

[74] Begum G., Venkateswara Rao J., Srikanth K. (2006). Oxidative stress and changes in locomotor behavior and gill morphology of *Gambusia affinis* exposed to chromium. Toxicol. Environ. Chem..

[75] Rochman C.M., Hentschel B.T., Teh S.J. (2014). Long-term sorption of metals is similar among plastic types: Implications for plastic debris in aquatic environments. PLoS ONE.

