# Supplementary material for: Exposure to Environmentally Relevant Concentrations of Polystyrene Microplastics Increases Hexavalent Chromium Toxicity in Aquatic Animals
Source: Toxics. 2022 Sep 26;10(10):563. doi: 10.3390/toxics10100563 (PMC9607387; doi:10.3390/toxics10100563)
Supplement: Supplementary file 1 [file toxics-10-00563-s001.zip › toxics-1868804-supplementary.pdf]

toxics MDPI

Supplementary Materials: Exposure to Environmentally Relevant Concentrations of Polystyrene Microplastics Increases Hexavalent Chromium Toxicity in Aquatic Animals

Jaehee Kim, Md. Niamul Haque, Somyeong Lee, Do-Hee Lee and Jae-Sung Rhee

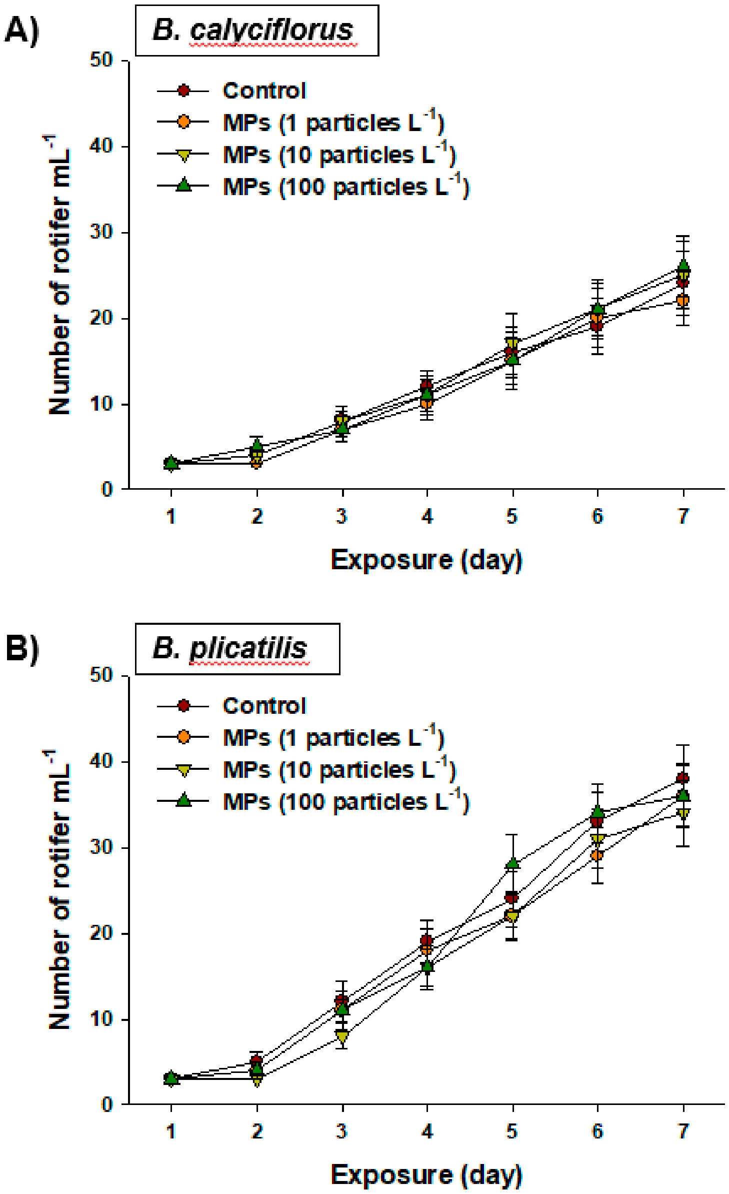

Figure S1. The number of rotifers upon MPs alone.

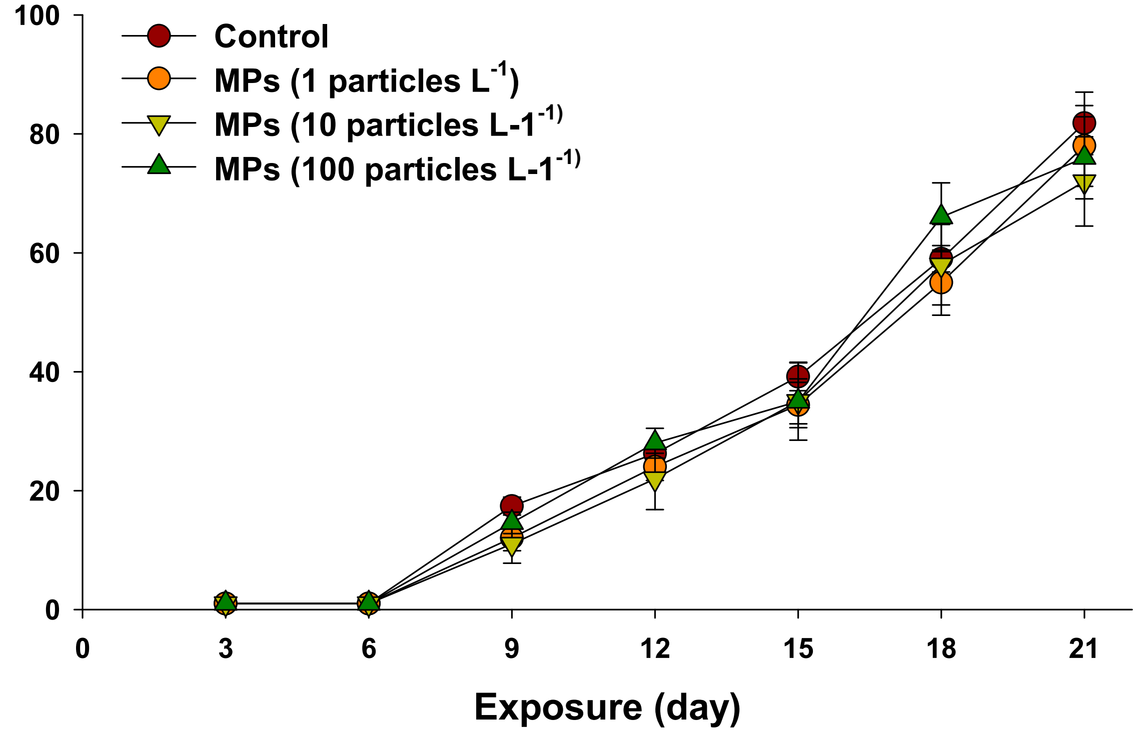

Figure S2. The number of daphnids upon MPs alone.

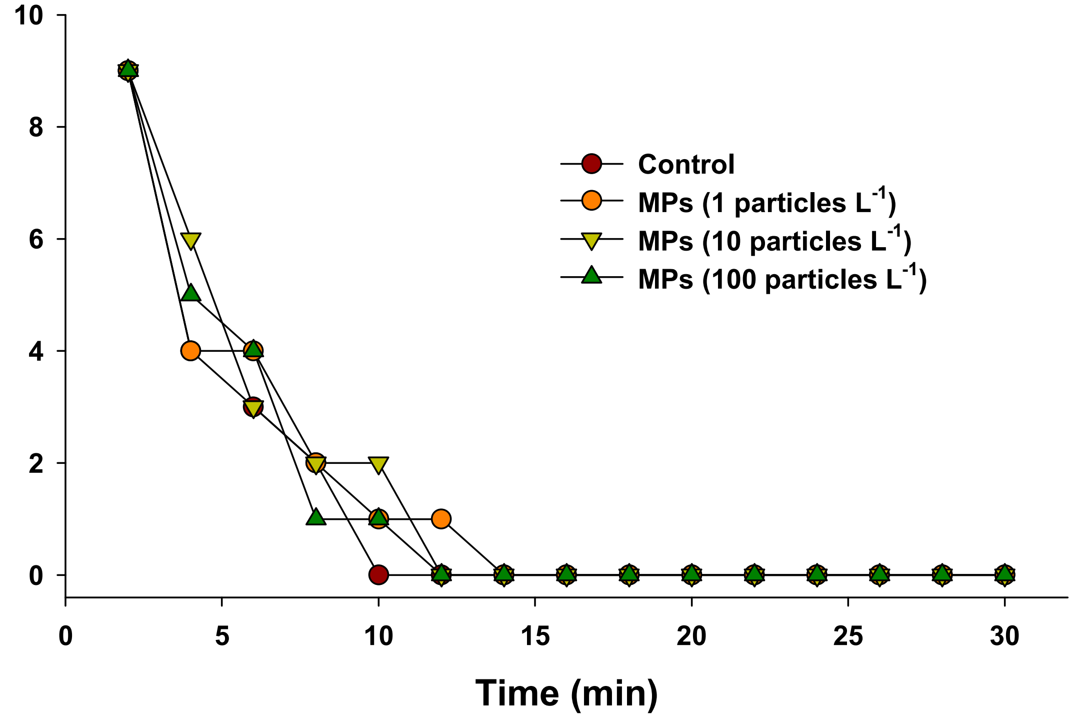

Figure S3. Burrowing activity of polychaete upon MPs alone.

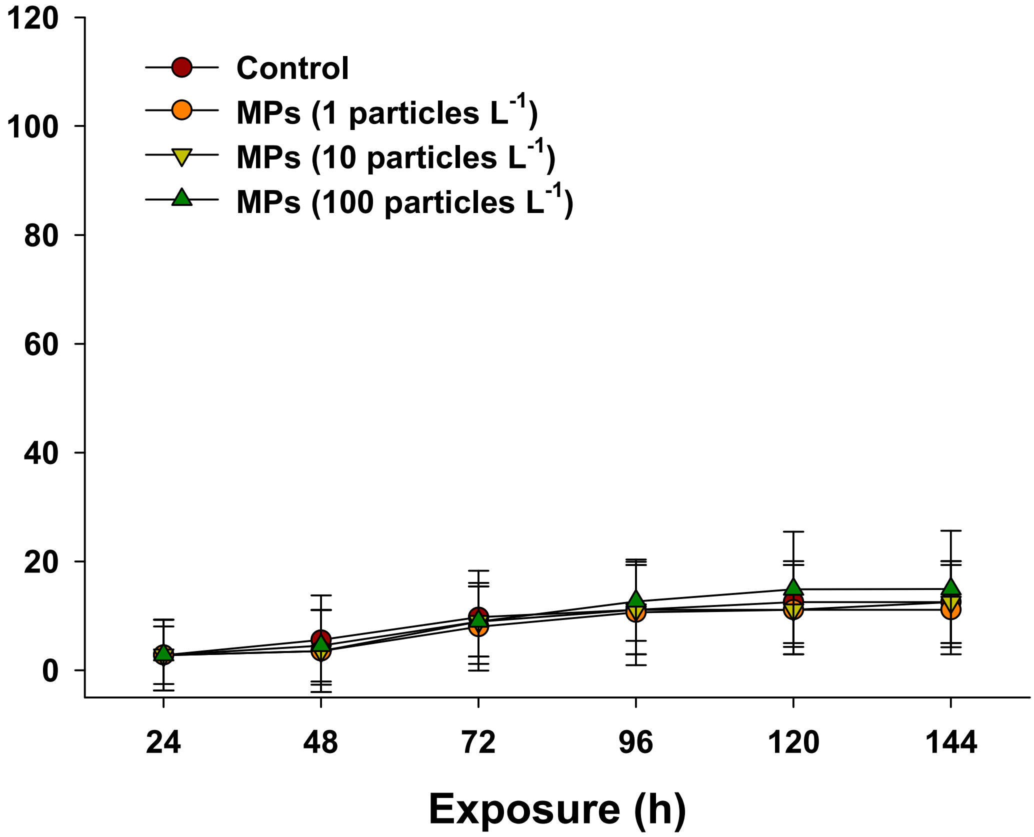

Figure S4. Mortality of zebrafish upon MPs alone.
